# Career adaptability under AI-related employment pressure: a multi-method study of relational structure and configurational pathways

**DOI:** 10.3389/fpsyg.2026.1923965

**Published:** 2026-09-09

**Authors:** Dan Chen, Jinling Wang

**Affiliations:** 1 School of Teacher Education, Weifang University, Weifang, China; 2 School of Languages and Media, Anhui University of Finance and Economics, Bengbu, China

**Keywords:** artificial intelligence-related employment pressure, career adaptability, configurational pathways, goal reengagement intention, outcome expectations, university students in the school-to-work transition

## Abstract

**Objective:**

This study examines the factors, relational structure, and exploratory configurational associations related to career adaptability among university students in the school-to-work transition under artificial intelligence-related employment pressure.

**Methods:**

Grounded primarily in Social Cognitive Career Theory and supplemented by Career Construction Theory, this study draws on questionnaire data from 564 Chinese university students and integrates structural equation modelling, mediation analysis, multigroup analysis, necessary condition analysis, and fuzzy-set qualitative comparative analysis.

**Results:**

Career adaptability was directly associated with multidimensional cognitive, behavioural, and contextual factors, as well as with indirect pathways involving outcome expectations and goal reengagement intention. However, the serial indirect effects were generally weak, indicating that career adaptability was not explained primarily by a single sequential cognition–action pathway. The overall relational structure was largely stable across undergraduate and postgraduate groups, with differences in the strength of only a few paths. Necessary condition analysis identified no single necessary condition with a substantial practical effect, whereas the exploratory configurational analysis identified multiple combinations of conditions associated with higher career adaptability, suggesting substitution and compensation among different resources. Artificial intelligence threat perception was not associated exclusively with poorer adaptation; under specific configurations of conditions, it coexisted with higher career adaptability.

**Conclusion:**

Career adaptability in an employment context shaped by artificial intelligence is not determined by a single factor; rather, it represents a complex adaptive structure jointly associated with multiple cognitive, behavioural, and contextual resources. By combining the explanation of the relationship between career cognition and action offered by Social Cognitive Career Theory with the multi-resource perspective on adaptation provided by Career Construction Theory, this study advances understanding of career adaptability in contexts where artificial intelligence-related pressure and opportunities for collaboration coexist. The findings suggest that career education and employment support in universities should not be confined to training in a single skill. Instead, they should integrate capability beliefs, career judgements, technology-related practice, goal adjustment, and external support to provide diverse career preparation pathways for students with different resource profiles.

## 1 Introduction

Against the backdrop of the continued penetration of artificial intelligence (AI) into the labour market, employment structures are undergoing profound transformations (Schmidt et al., 2025). Automation-driven job restructuring, changing skill requirements, and the reconfiguration of occupational boundaries have created an increasingly complex career decision-making environment for university students (Martin et al., 2025). In China in particular, where the number of university graduates continues to grow and competition for employment remains intense, AI is further reshaping students’ judgements of career opportunities, capability requirements, and future development directions. Examining university students’ career adaptability in an AI employment context is therefore important for understanding career preparation and development amid technological change.

Previous research has examined the employment implications of AI at the levels of labour-market structures, career cognition, and individual behaviour, covering general workers, organisational employees, university students, and the labour market as a whole (Okulich-Kazarin et al., 2024; Korayim et al., 2025; Ahn et al., 2025). By comparison, university students in the school-to-work transition have received relatively limited attention (Pavone, 2026; Xu et al., 2025). Although this group has yet to establish stable occupational roles, its members already face practical tasks involving skill updating, career choice, and employment preparation. Their career adaptability is thus manifested primarily in judgements about the future occupational environment, their state of preparedness, and their propensity to act, giving it stage-specific characteristics distinct from those of employed populations (Tomičić et al., 2025).

Meanwhile, the literature presents two complementary yet relatively separate explanatory strands. One emphasises the consequences of technological threat, including job displacement, skill devaluation, and employment anxiety (Yin, 2025); the other increasingly focuses on the developmental value of human–artificial intelligence collaboration, capability augmentation, and emerging career opportunities (Xu Y. W. et al., 2024; Lu and Kim, 2025). These two strands, respectively, reveal the pressure-inducing and collaborative attributes of AI. However, a unified career-development explanation is still lacking for how both attributes jointly inform individuals’ career judgements and how capability beliefs, technology-related perceptions, actual engagement, and external support are associated with career adaptability. In particular, when changes in jobs and skills reduce the feasibility of an existing career goal, whether individuals can redirect their efforts and remain engaged has yet to be adequately incorporated into research on AI and employment.

Furthermore, higher career adaptability may not correspond to a single configuration of conditions. Previous research has found that human, social, and psychological capital can be combined in multiple ways associated with higher career adaptability and that a deficiency in one form of capital may be compensated for by another (Xu Q. et al., 2024). Some individuals may therefore rely primarily on strong capability beliefs and positive developmental expectations, whereas others may maintain career preparedness under conditions of high perceived risk through technology-related engagement, goal adjustment, or external support. Methodologically, analyses of average net associations mainly examine the independent contribution of individual variables and cannot readily reveal the joint operation of multiple conditions, equifinality, or substitution and compensation among conditions (Fiss, 2011). Nor can they directly determine whether a factor constitutes a necessary condition without which the outcome cannot occur (Dul, 2016).

Accordingly, this study adopts the career self-management model of Social Cognitive Career Theory (SCCT) as its primary framework for explaining the associations among capability beliefs, contextual conditions, outcome expectations, and career-related action. It also draws on Career Construction Theory (CCT) to define career adaptability and its character as a resource for coping with career change. On this basis, the study advances the literature in three ways. First, it extends research on AI and employment from separate attention to technological threats or collaborative opportunities to the issue of career adaptation in a context where the two coexist. It focuses specifically on university students—principally senior undergraduates and postgraduates—who are in or approaching the school-to-work transition. Second, within the SCCT framework, it distinguishes the cognitive appraisal represented by outcome expectations from the action regulation represented by goal reengagement intention. By incorporating redirection following obstruction of an existing career goal into the AI employment context, it refines the association between career cognition and action regulation. Third, by integrating structural equation modelling (SEM), multigroup analysis (MGA), necessary condition analysis (NCA), and fuzzy-set qualitative comparative analysis (fsQCA), it examines average associations, differences by educational stage, the necessity constraints imposed by individual conditions, and combinations of multiple conditions, respectively. This approach supplements the limited capacity of a single linear analysis to explain the diversity of pathways to career adaptability.

On the basis of the preceding analysis, this study addresses the following two core research questions:

*RQ1:* Under AI-related employment pressure, which contextual, cognitive, and behavioural factors are significantly associated with career adaptability among university students in the school-to-work transition?

*RQ2:* Are these factors associated with higher career adaptability through multiple configurations of conditions?

## 2 Literature review

### 2.1 Theoretical foundations of career adaptability among university students in the school-to-work transition in the AI employment context

As AI continues to transform job structures, skill requirements, and occupational boundaries, university students must not only assess the employment risks that technological change may create but also recognise the developmental opportunities afforded by human–AI collaboration, capability enhancement, and emerging occupational activities (Bozkurt and Gursoy, 2025). Unlike variables such as career identity, technology acceptance, or job-search anxiety, which focus on particular psychological or behavioural responses, career adaptability emphasises an individual’s preparedness and capacity for adjustment when confronting career tasks, role transitions, and environmental uncertainty. Savickas (1997) defined career adaptability as an important adaptive construct integrating individual career development with environmental change. Savickas and Porfeli (2012) subsequently operationalised it as a psychosocial resource comprising career concern, career control, career curiosity, and career confidence, and developed the Career Adapt-Abilities Scale accordingly. Career adaptability is therefore particularly suitable for examining individuals’ overall state of career development in the AI employment context (Lent and Brown, 2013).

Existing research offers different but complementary perspectives on the employment implications of AI. On the one hand, AI threat perception may be associated with employment anxiety, identity threat, technology avoidance, and hesitation to use technology, suggesting that anticipated job displacement and skill devaluation may weaken individuals’ sense of security regarding their future careers (Wu et al., 2026; Kitchens and Meier, 2026). On the other hand, by extending task capabilities, improving work efficiency, and creating new occupational activities, AI may be positively associated with technology-related engagement, career preparation, and capability enhancement (Lu and Kim, 2025; Santos-Jaén et al., 2025; Li and Li, 2026). Some studies have also found that technological threat does not necessarily correspond only to avoidance; it may heighten individuals’ appreciation of creativity, professional competence, and the value of career differentiation (Gamez-Djokic et al., 2026). These not entirely consistent findings indicate that AI-related threats and developmental opportunities are not mutually exclusive and may jointly inform individuals’ judgements of their career environment.

However, existing studies generally examine technological threat, attitudes towards AI, intention to use AI, learning engagement, or employment anxiety separately. Few have situated capability beliefs, risk perceptions, perceptions of human–AI collaboration, actual use engagement, and external support within a single career-development framework (Rogers, 1975; Floyd et al., 2000; Xia et al., 2026). Technological change may also affect not only individuals’ judgements of future career outcomes but also the feasibility of their existing career goals, confronting them with the question of whether to redirect and reinvest their efforts. Explaining the employment implications of AI solely in terms of threat coping or technology acceptance is therefore insufficient to show how different cognitive, behavioural, and contextual resources are jointly associated with career adaptability.

The career self-management model of SCCT provides the principal framework for integrating these factors (Lent et al., 1994). The theory emphasises the interrelationships among self-efficacy, outcome expectations, contextual supports and barriers, and career-related action, thereby explaining how individuals manage career tasks and respond to career change (Lent and Brown, 2013). In the AI employment context, AI self-efficacy captures an individual’s belief in their capability to use AI to address learning and career-related tasks. AI threat perception, perceived human–AI collaboration, and perceived employment support provide information about risk, opportunities for collaboration, and external resources, respectively, while AI use engagement reflects an individual’s actual exposure to and active involvement with the technology. SCCT can therefore integrate capability judgements, environmental information, career expectations, and propensities for action in the AI context within a unified relational structure.

CCT, in turn, provides the theoretical basis for the substantive meaning of career adaptability as the outcome (Voigt and Strauss, 2025). The theory regards career adaptability as a psychosocial resource that individuals can mobilise when confronting career tasks, transitions, and uncertainty. It emphasises that career development does not follow a single pathway but requires continual adjustment in response to environmental change (Savickas and Porfeli, 2012). Accordingly, SCCT primarily explains the associations among individual and contextual factors, career cognition, and propensities for action, whereas CCT defines career adaptability and its multi-resource character. Combining the two enables career adaptation in the AI employment context to be explained in terms of both its relational structure and individuals’ responses to career change.

### 2.2 Cognitive appraisal and action regulation in career adaptability

Within the career self-management model of SCCT, outcome expectations are an important cognitive variable linking capability and contextual information to career-related action. They reflect an individual’s judgement of the career outcomes that a particular action may produce. Outcome expectations differ from self-efficacy: self-efficacy concerns whether individuals believe that they can perform an action (Bandura, 1977), whereas outcome expectations concern the benefits and risks associated with taking that action and its implications for career development (Lent and Brown, 2013). In the AI employment context, individuals may judge that learning and using AI will enhance their employment competitiveness while also considering the costs of investing in new skills, the risk of job displacement, and uncertainty about achieving their career goals.

Expectancy–value theory further clarifies the meaning of outcome expectations. It proposes that an individual’s choice of action depends not only on the likelihood that an outcome will occur but also on the value of that outcome and its anticipated costs (Eccles and Wigfield, 2002). Research indicates that university students’ appraisals of the value, probability of success, and risk costs of learning about AI are closely associated with their intentions to learn and use it (Cheng, 2025). Outcome expectations in this study therefore do not represent a merely positive belief. Rather, they constitute an integrated judgement that takes account of the career opportunities, competitive advantages, and potential risks associated with AI.

Positive outcome expectations, however, do not imply that an individual will always continue to pursue an established career goal. AI may change the task content and capability requirements of existing jobs, reducing the feasibility of some career goals. Under such circumstances, adaptive action entails not only persistence but also reassessing goals, redirecting one’s career, and investing resources in viable alternatives. The goal-reengagement perspective proposed by Wrosch et al. (2003) suggests that the ability to identify alternative goals and remain engaged when an existing goal is obstructed is an important component of self-regulation. Recent research has likewise found that the capacity for goal reengagement can shape individuals’ judgements of their career development after exposure to AI (Voigt and Strauss, 2025).

Outcome expectations and goal reengagement intention thus represent cognitive appraisal and action regulation, respectively, in the process of career adaptation. The former concerns the career outcomes that may follow AI-related actions, whereas the latter concerns whether individuals are willing to change direction and remain engaged when their existing career pathway is constrained. Incorporating goal reengagement into the SCCT framework extends the career self-management model, which mainly explains capability beliefs, outcome expectations, and actions directed towards established goals, so that it also encompasses situations in which technological change reduces the feasibility of an existing goal. Although outcome expectations and goal reengagement intention are interrelated, they do not necessarily form a fixed linear sequence. Individuals may maintain their established direction while holding positive career expectations, or they may proactively adjust their goals in response to high perceived risk. Each may also be associated with career adaptability in conjunction with AI self-efficacy, AI use engagement, and perceived employment support. Accordingly, this study treats outcome expectations and goal reengagement intention as cognitive and action-oriented variables with distinct functions and examines their independent associations, statistical indirect relationships, and participation with other conditions in different resource configurations.

## 3 Research model and research design

### 3.1 Research model and hypotheses

On the basis of the preceding theoretical analysis, this study uses the career self-management model of SCCT as its primary framework and combines it with CCT’s definition of career adaptability to develop a research model comprising antecedent factors, outcome expectations, goal reengagement intention, and career adaptability. AI self-efficacy, AI threat perception, perceived human–AI collaboration, AI use engagement, and perceived employment support serve as antecedent variables. Outcome expectations and goal reengagement intention represent cognitive appraisal and action regulation, respectively, while career adaptability is the outcome variable. Because the five antecedent variables have distinct theoretical meanings, their respective relationships with outcome expectations, goal reengagement intention, and career adaptability are developed below.

#### 3.1.1 AI self-efficacy and variables related to career adaptability

AI self-efficacy reflects individuals’ judgements of their capability to learn, master, and use AI tools to complete career preparation tasks. According to SCCT, stronger capability beliefs are generally associated with more positive judgements of the application value and career benefits of AI. They may also give individuals greater confidence to learn new skills, explore alternative jobs, and redirect their careers when changes in jobs or skills disrupt their original career plans (Lent et al., 1994; Lent and Brown, 2013). Related research also indicates that AI self-efficacy is closely associated with the perceived value of technology use, employment confidence, and career-development expectations (Zhang et al., 2026). AI self-efficacy may therefore be associated with both more positive outcome expectations and stronger goal reengagement intention, while also constituting an important capability resource that helps individuals respond to career change and remain prepared.

*H1a:* AI self-efficacy is significantly and positively associated with outcome expectations.

*H2a:* AI self-efficacy is significantly and positively associated with goal reengagement intention.

*H4a:* AI self-efficacy is significantly and positively associated with career adaptability.

#### 3.1.2 AI threat perception and variables related to career adaptability

AI threat perception reflects individuals’ subjective judgements of technological risks such as job displacement, skill devaluation, and intensified employment competition. On the one hand, previous research has found that higher AI threat perception may be accompanied by fear, identity threat, and hesitation to use technology (Xu Y. W. et al., 2024; Kitchens and Meier, 2026). On the other hand, research on protection motivation suggests that risk perception may prompt individuals to reassess environmental demands and their own coping capabilities and may increase their awareness of the need for capability development and career preparation (Rogers, 1975; Floyd et al., 2000). During the school-to-work transition, greater sensitivity to AI-related risks may lead students to attach more importance to the benefits of technology learning and career adjustment and to reassess their development direction when an existing goal becomes less feasible (Gamez-Djokic et al., 2026). This study therefore expects AI threat perception to reflect not only employment pressure but also a positive association with outcome expectations, goal reengagement intention, and career adaptability.

*H1b:* AI threat perception is significantly and positively associated with outcome expectations.

*H2b:* AI threat perception is significantly and positively associated with goal reengagement intention.

*H4b:* AI threat perception is significantly and positively associated with career adaptability.

#### 3.1.3 Perceived human–AI collaboration and variables related to career adaptability

Perceived human–AI collaboration reflects the extent to which individuals regard AI as a resource for task assistance and capability extension. A higher level of perceived human–AI collaboration may direct greater attention to the value of AI for information processing, task completion, and capability development and may be associated with more positive career outcome expectations (Li and Li, 2026). When the tasks within an existing job or a career pathway change, individuals who regard AI as a collaborative resource may also be more likely to use new technological tools to explore alternative tasks and career opportunities, thereby sustaining their investment in action after adjusting their goals (Kong et al., 2023). Perceived human–AI collaboration is closely associated with technology-related engagement, capability enhancement, and proactive career adjustment (Santos-Jaén et al., 2025). Perceived human–AI collaboration may therefore be positively associated with outcome expectations, goal reengagement intention, and career adaptability.

*H1c:* Perceived human–AI collaboration is significantly and positively associated with outcome expectations.

*H2c:* Perceived human–AI collaboration is significantly and positively associated with goal reengagement intention.

*H4c:* Perceived human–AI collaboration is significantly and positively associated with career adaptability.

#### 3.1.4 AI use engagement and variables related to career adaptability

AI use engagement captures the frequency, breadth, and continuity of individuals’ use of AI tools in learning, job seeking, and career preparation. Sustained engagement with technology can provide direct experience and task feedback, enabling individuals to make more concrete judgements about the potential value of AI for improving efficiency, developing skills, and expanding career opportunities. It may therefore be associated with more clearly defined outcome expectations (Lu and Kim, 2025). Diverse use experiences may also increase individuals’ understanding of new tasks and career directions, giving them a wider range of possible actions when their existing career plan is constrained. Previous research likewise indicates that actual AI use and engagement in learning are positively associated with technological adaptation, innovative behaviour, and the development of employability (Ran and Feng, 2026; Shen et al., 2026). AI use engagement may accordingly be positively associated with outcome expectations, goal reengagement intention, and career adaptability.

*H1d:* AI use engagement is significantly and positively associated with outcome expectations.

*H2d:* AI use engagement is significantly and positively associated with goal reengagement intention.

*H4d:* AI use engagement is significantly and positively associated with career adaptability.

#### 3.1.5 Perceived employment support and variables related to career adaptability

Perceived employment support refers to individuals’ overall evaluation of the employment information, career guidance, capability training, and practical opportunities provided by their university. SCCT regards contextual support as an important external resource shaping career judgements and career-related action. Adequate employment support may reduce the cost of obtaining occupational information and developing skills and may be associated with more positive judgements of future career opportunities and the returns to action (Lent et al., 2000). When changes in the employment environment affect an existing career goal, support in the form of courses, counselling, internships, and employment information can also help individuals identify alternative jobs, supplementary skills, and new development directions, thereby sustaining engagement after goal adjustment (Carvalho et al., 2023). University career education and employment support are also closely related to students’ career confidence, employment preparation, and employability (Barachino et al., 2025). Perceived employment support may therefore be positively associated with outcome expectations, goal reengagement intention, and career adaptability.

*H1e:* Perceived employment support is significantly and positively associated with outcome expectations.

*H2e:* Perceived employment support is significantly and positively associated with goal reengagement intention.

*H4e:* Perceived employment support is significantly and positively associated with career adaptability.

#### 3.1.6 Outcome expectations, goal reengagement intention, and career adaptability

Outcome expectations reflect individuals’ integrated judgements of the career benefits, development opportunities, and potential costs associated with AI-related actions. Expectancy–value theory proposes that when individuals expect an action to produce a valued outcome, they are generally more willing to invest in subsequent action (Eccles and Wigfield, 2002). More positive outcome expectations may therefore be associated with individuals’ willingness to continue exploring alternative directions after a career goal is constrained, as well as with their preparedness and adjustment when confronting career change (Korkmaz and Yam, 2023). Goal reengagement intention reflects the propensity to seek alternative goals and reallocate resources for action when an existing goal becomes less feasible. It is an important self-regulatory resource for coping with career transitions and uncertainty (Wrosch et al., 2003). From the perspective of CCT, adjusting one’s development direction in response to environmental change while remaining engaged is also an important manifestation of career adaptability (Savickas and Porfeli, 2012). This study therefore expects outcome expectations to be positively associated with goal reengagement intention and both variables to be positively associated with career adaptability.

*H3:* Outcome expectations are significantly and positively associated with goal reengagement intention.

*H5:* Outcome expectations are significantly and positively associated with career adaptability.

*H6:* Goal reengagement intention is significantly and positively associated with career adaptability.

On the basis of these relationships, the study further tests the statistical indirect relationships of outcome expectations and goal reengagement intention between the five antecedent variables and career adaptability, as well as the serial indirect relationships involving both variables. Because the five antecedents represent distinct capability, risk, collaborative, behavioural, and contextual resources, the study does not assume that all indirect pathways have the same strength of association.

### 3.2 Research methods

#### 3.2.1 Participants and sampling method

This study understands the “school-to-work transition” as a continuous developmental stage in which individuals gradually move from student to occupational roles and progress from general career exploration to career choice, career preparation, and labour-market entry. On this basis, the study surveyed university students in China, focusing on third- and fourth-year undergraduates and postgraduate students, including master’s and doctoral students. Compared with students in the first two undergraduate years, third- and fourth-year students have entered the later stage of higher education. Their learning tasks gradually shift from foundational coursework to the integration of professional capabilities, internships and practical experience, and decisions about post-graduation destinations; career choice has therefore become a practical task requiring concrete judgement and preparation. Postgraduate education has a more explicit emphasis on specialisation and career direction, and postgraduate students likewise need to address occupational positioning, skill matching, and employment choices after completing their degrees. Although the sample covers university students at different stages of study, it is therefore concentrated among senior undergraduates and postgraduates whose career-development tasks have shifted from general exploration to concrete employment decisions and preparation.

Convenience sampling was used for three reasons. First, the principal purpose of the study was to examine the relational structure and configurational pathways among variables rather than to estimate population parameters, making convenience sampling appropriate for the present test of theoretical relationships. Second, participants needed experience using AI tools, and the study focused on senior undergraduates and postgraduate students; convenience sampling facilitated effective access to eligible respondents under limited resource conditions. Third, an online questionnaire platform enabled the study to reach students from different regions, types of institution, and disciplinary backgrounds, thereby increasing sample diversity to some extent.

#### 3.2.2 Questionnaire and scale design

A self-report questionnaire was used for measurement, and all items were rated on a five-point Likert scale (1 = “does not describe me at all”, 5 = “describes me completely”). The questionnaire contained eight latent variables, with 33 measurement items retained in the final instrument. Items for each variable were adapted primarily from theoretical frameworks and measurement instruments in the existing literature. During item screening, four items with standardised factor loadings below 0.60 were removed on the basis of the confirmatory factor analysis: one item measuring AI self-efficacy, two measuring AI threat perception, and one measuring perceived human–AI collaboration. Model fit improved after their removal (Δχ^2^/df = 2.10, ΔRMSEA = 0.006, ΔCFI = 0.008), without materially compromising the content validity of any dimension. The retained items and their sources are presented in Table 1.

**Table 1:** Core measurement items.

Variable	Measurement item	References
Artificial intelligence self-efficacy (AISE)	I am confident that I can master the use of AI tools relevant to learning or job seeking.	Chen et al. (2026)
	Even when encountering a new AI tool, I can learn how to use it relatively quickly.	
	I believe that I can apply AI tools effectively in my career preparation.	
	When problems arise while I am using AI tools, I can usually find a solution.	
Artificial intelligence threat perception (AITP)	I worry that advances in AI will weaken my competitive advantage in employment.	Wu et al. (2026) and Yin (2025)
	I believe that AI is intensifying employment competition among university graduates.	
	I worry that AI will replace some jobs that would otherwise be suitable for university graduates.	
	Advances in AI make me feel more uncertain about my future career prospects.	
Perceived human–artificial intelligence collaboration (PHAC)	I believe that AI can be an effective tool in my career preparation.	Li and Li (2026) and Zahs and Schmodde (2026)
	When completing learning, job-search, or career-planning tasks, I am willing to collaborate with AI on some of the work.	
	I believe that human–AI collaboration can improve task-completion efficiency.	
	I tend to regard AI as a collaborative resource for career development rather than merely as a threat.	
Artificial intelligence use engagement (AIUE)	I frequently use AI tools to support learning, job seeking, or career preparation.	Lu and Kim (2025) and Segbenya et al. (2023)
	I proactively try to apply AI to different types of tasks.	
	I use AI in a variety of contexts during career preparation.	
	I am willing to continue investing time and effort in learning new AI functions.	
Perceived employment support (PES)	My university provides the support students need to address employment challenges in the age of AI.	Lent and Brown (2013) and Zhang et al. (2026)
	The courses, lectures, or activities provided by my university help improve my career preparedness.	
	I can obtain useful information about AI and employment from my university.	
	My university’s career guidance or support services increase my confidence in facing the future labour market.	
Outcome expectations (OE)	Developing AI-related capabilities will help me obtain better employment opportunities.	Lent and Brown (2013) and Cheng (2025)
	Proactively adapting to changes brought about by AI will enhance my career competitiveness.	
	Learning to work collaboratively with AI will benefit my career development.	
	Continually developing my capabilities in an AI environment will help me achieve my career goals.	
Goal reengagement intention (GRI)	Even if my original career plan is disrupted, I am willing to plan a new development direction.	Wrosch et al. (2003) and Voigt and Strauss (2025)
	When the employment environment changes, I am willing to continue investing time and effort in seeking new opportunities.	
	Even if I encounter setbacks while seeking work, I will adjust my goals and continue my efforts.	
	When my original career pathway becomes difficult to realise, I can proactively seek alternatives.	
Career adaptability (CA)	I can adjust my career plan promptly in response to a changing employment environment.	Savickas and Porfeli (2012)
	I am confident in my ability to address uncertainty and challenges in my future career development.	
	I can proactively prepare for future career opportunities.	
	I can usually adapt relatively quickly to new employment requirements.	
	Even when the employment environment changes, I can identify a development direction that suits me.	

For scales and theoretical concepts originating in English, a translation–back-translation procedure was followed to produce the Chinese version. First, two researchers with bilingual Chinese–English backgrounds independently translated the English scales into Chinese and integrated their translations into a preliminary version through discussion. Another researcher who had not participated in the initial translation then translated the Chinese version back into English. The back-translation was compared with the original, and items exhibiting semantic discrepancies were revised until agreement was reached. The research team subsequently adapted the wording of the items to the context of AI-related employment pressure and the school-to-work transition. All adaptations retained the meanings of the original concepts.

#### 3.2.3 Data collection and sample screening

Data were collected through the Chinese online survey platform Wenjuanxing between 20 March and 18 April 2026. The questionnaire was distributed through three channels. First, university career guidance centres and departmental counsellors circulated it directly to senior undergraduates and postgraduate students. Second, it was posted in university social-media groups, including year-group, graduating-student, and postgraduate groups, and forwarded by class contacts. Third, respondents were invited to recommend the survey to students in the same year or discipline through snowball referrals. This approach covered different institutional levels and disciplinary backgrounds and increased the diversity of sample sources. Respondents came from multiple provinces in eastern, northern, and southern China and in the central and western regions. They attended comprehensive, teacher-training, and science and engineering universities and represented science and engineering, the humanities and social sciences, economics, management and law, and other disciplinary categories. The inclusion criteria were: (1) being an undergraduate or postgraduate student at a Chinese university, with particular coverage of third- and fourth-year undergraduates and postgraduate students; (2) having experience using AI tools; and (3) completing the questionnaire and passing the quality checks. Data-quality controls included an attention-check item, the exclusion of questionnaires completed in less than 120 s, logical-consistency checks, and screening for duplicate IP addresses.

Before the main survey, a pilot test was conducted in early March 2026 with 45 third- and fourth-year undergraduates and postgraduate students to evaluate item comprehensibility and scale reliability. The pilot sample’s mean completion time was 3.2 min, and Cronbach’s α for the latent variables ranged from 0.85 to 0.89. The wording of three items was refined in response to participant feedback, and the pilot data were not included in the main analysis. A total of 628 questionnaires were collected in the main survey. After screening according to the prespecified criteria (Table 2), 564 valid responses remained.

**Table 2:** Sample-screening procedure.

Screening step	Exclusion criterion	Excluded	Retained
Questionnaires initially collected	—	—	628
Abnormally short completion time	Completed in <120 s	20	608
Failed attention check	Incorrect response	15	593
Experience using AI tools	Responded “No”	5	588
Logical inconsistency	Inconsistent responses to positive and reverse items within a dimension	8	580
Consecutive invariant responses	Repeated selection of the same option with extremely low variance	8	572
Duplicate IP address	Multiple submissions from the same IP; first retained	8	564

The study followed the ethical principles of the Declaration of Helsinki and was approved by the Research Ethics Committee of Weifang University (approval no. WFY2026-045). Before completing the questionnaire, all participants read an informed-consent statement on the opening page explaining the study purpose, use of the data, anonymisation procedures, and voluntary nature of participation. Only those who clicked “Agree” could proceed to the questionnaire. Participation was entirely voluntary, the data were used solely for academic research, and all responses were anonymised before analysis. Data analyses were conducted using SPSS 26.0, Amos 24.0, R 4.5.3 with the NCA 5.0.0 package, and fsQCA 3.0.

## 4 Data analysis and results

### 4.1 Sample characteristics and descriptive statistics

The sample included undergraduates from different years of study and postgraduate students. Third- and fourth-year undergraduates accounted for 72.4% of the sample and postgraduate students and above for 23.9%; together, these two groups represented 96.3% of the sample. The sample was thus concentrated in the later stages of higher education and the school-to-work transition and was highly relevant to the employment context. All respondents included in the analyses had experience using AI tools. The gender distribution was relatively balanced (55.3% men and 44.7% women), and most participants were 18–22 years old (64.3%). The sample also exhibited some diversity in disciplinary background, practical experience, and participation in AI-related courses. Most respondents were either students not currently employed or were seeking work.

The means of the core variables ranged from 3.465 to 3.631 and were all above the scale midpoint. Standard deviations ranged from 1.021 to 1.230, indicating a degree of individual variation in the variables (Table 3).

**Table 3:** Descriptive statistics for the core variables.

Variable	Minimum	Maximum	Mean	SD
Artificial intelligence self-efficacy	1.00	5.00	3.6175	1.0975
Artificial intelligence threat perception	1.00	5.00	3.5496	1.1338
Perceived human–artificial intelligence collaboration	1.00	5.00	3.5997	1.0951
Artificial intelligence use engagement	1.00	5.00	3.4650	1.0598
Perceived employment support	1.00	5.00	3.5869	1.0205
Outcome expectations	1.00	5.00	3.5727	1.1222
Goal reengagement intention	1.00	5.00	3.4752	1.2301
Career adaptability	1.00	5.00	3.6312	1.1288

### 4.2 Data quality assessment

#### 4.2.1 Data distribution and suitability tests

Before the main analyses, the data were examined for missing values, normality, outliers, and multicollinearity. None of the 33 measurement items contained missing values, and no imputation was therefore performed. The absolute skewness values of the items ranged from 0.216 to 0.784, and their absolute kurtosis values ranged from 0.434 to 1.186. On the basis of standardised scores, no univariate outlier exceeded an absolute value of 3.29, indicating no severe univariate non-normality. Mardia’s test of multivariate kurtosis was significant (multivariate kurtosis coefficient = 52.590, critical ratio = 12.993, *p* < 0.001), indicating that the data did not satisfy the assumption of multivariate normality. Given the large sample and the absence of severe univariate non-normality in the items, maximum likelihood estimation was nevertheless used for the subsequent SEM. To address multivariate non-normality, the main SEM parameter estimates were additionally evaluated using 5,000 bootstrap resamples, with statistical significance assessed using bias-corrected 95% confidence intervals.

Mahalanobis distance was then used to identify multivariate outliers. Five cases exceeded the chi-square critical value at *p* < 0.001, representing 0.89% of the sample. Inspection showed that all item responses in these cases fell within the valid scale range and revealed no obvious logical contradictions or extreme univariate values; the cases were therefore retained. Variance inflation factors ranged from 1.361 to 1.766, all below the threshold of 5, indicating no serious multicollinearity. The Kaiser–Meyer–Olkin value was 0.935, and Bartlett’s test of sphericity was significant (χ^2^ = 11,152.330, *p* < 0.001), demonstrating sufficient intercorrelations among the variables and the suitability of the data for subsequent factor analysis.

#### 4.2.2 Common method Bias

Because all variables were measured using a self-report questionnaire, common method bias was addressed through both procedural controls and statistical tests. Procedural measures included anonymous completion, mixed item ordering, and an attention-check item. Statistically, Harman’s single-factor test first showed that the first factor explained 37.568% of the variance, below the 50% threshold (Podsakoff and Organ, 1986).

A common latent factor test was subsequently performed (Table 4) by adding to the original factor measurement model a common latent method factor loading on all measurement items. Changes in model-fit indices were limited (ΔCFI = 0.010, ΔTLI = 0.008, and a reduction of 0.004 in RMSEA), indicating that the common method factor had no clear substantive effect on the measurement model. Taken together, these tests provided no evidence that common method bias posed a serious threat to the main results.

**Table 4:** Common latent method factor model.

Model	χ^2^	df	χ^2^/df	CFI	TLI	RMSEA	RMSEA 90% CI
Factor measurement model	989.972	467	2.120	0.957	0.952	0.042	[0.039, 0.046]
Common latent method factor model	789.181	434	1.818	0.967	0.960	0.038	[0.034, 0.042]
Structural model	912.065	467	1.953	0.959	0.954	0.041	[0.037, 0.045]

#### 4.2.3 Measurement model

As shown in Table 5, Cronbach’s α coefficients for the latent variables ranged from 0.841 to 0.885, and composite reliability (CR) ranged from 0.879 to 0.914; all exceeded the recommended threshold of 0.70, indicating good internal consistency. Standardised factor loadings ranged from 0.693 to 0.912, indicating acceptable convergent validity.

**Table 5:** Reliability and convergent-validity results.

Variable	Cronbach’s α	No. of items	Factor-loading range	CR	AVE
Artificial intelligence self-efficacy	0.878	4	0.704–0.912	0.903	0.699
Artificial intelligence threat perception	0.850	4	0.730–0.811	0.888	0.665
Perceived human–artificial intelligence collaboration	0.872	4	0.706–0.890	0.900	0.692
Artificial intelligence use engagement	0.863	4	0.737–0.846	0.905	0.704
Perceived employment support	0.841	4	0.693–0.834	0.879	0.645
Outcome expectations	0.865	4	0.738–0.807	0.901	0.695
Goal reengagement intention	0.876	4	0.769–0.838	0.907	0.709
Career adaptability	0.885	5	0.714–0.815	0.914	0.680

Discriminant validity was assessed in two ways. First, as shown in Table 6, the square root of the average variance extracted (AVE) for each latent variable exceeded its correlations with all other variables, satisfying the Fornell–Larcker criterion. Second, heterotrait–monotrait ratios (HTMT) ranged from 0.359 to 0.692, all below the threshold of 0.85. These results collectively indicated good discriminant validity among the latent variables.

**Table 6:** Discriminant-validity results.

Variable	AISE	AITP	PHAC	AIUE	PES	OE	GRI	CA
AISE	**0.836**							
AITP	0.459	**0.816**						
PHAC	0.406	0.514	**0.832**					
AIUE	0.428	0.418	0.397	**0.839**				
PES	0.366	0.492	0.403	0.347	**0.803**			
OE	0.546	0.574	0.497	0.497	0.516	**0.834**		
GRI	0.527	0.520	0.529	0.514	0.506	0.594	**0.842**	
CA	0.548	0.694	0.570	0.523	0.596	0.688	0.651	**0.825**

Bold diagonal values are the square roots of AVE; values below the diagonal are correlations between variables. AISE, AI self-efficacy; AITP, AI threat perception; PHAC, perceived human–AI collaboration; AIUE, AI use engagement; PES, perceived employment support; OE, outcome expectations; GRI, goal reengagement intention; CA, career adaptability.

### 4.3 Structural model analysis

**Figure 1:**
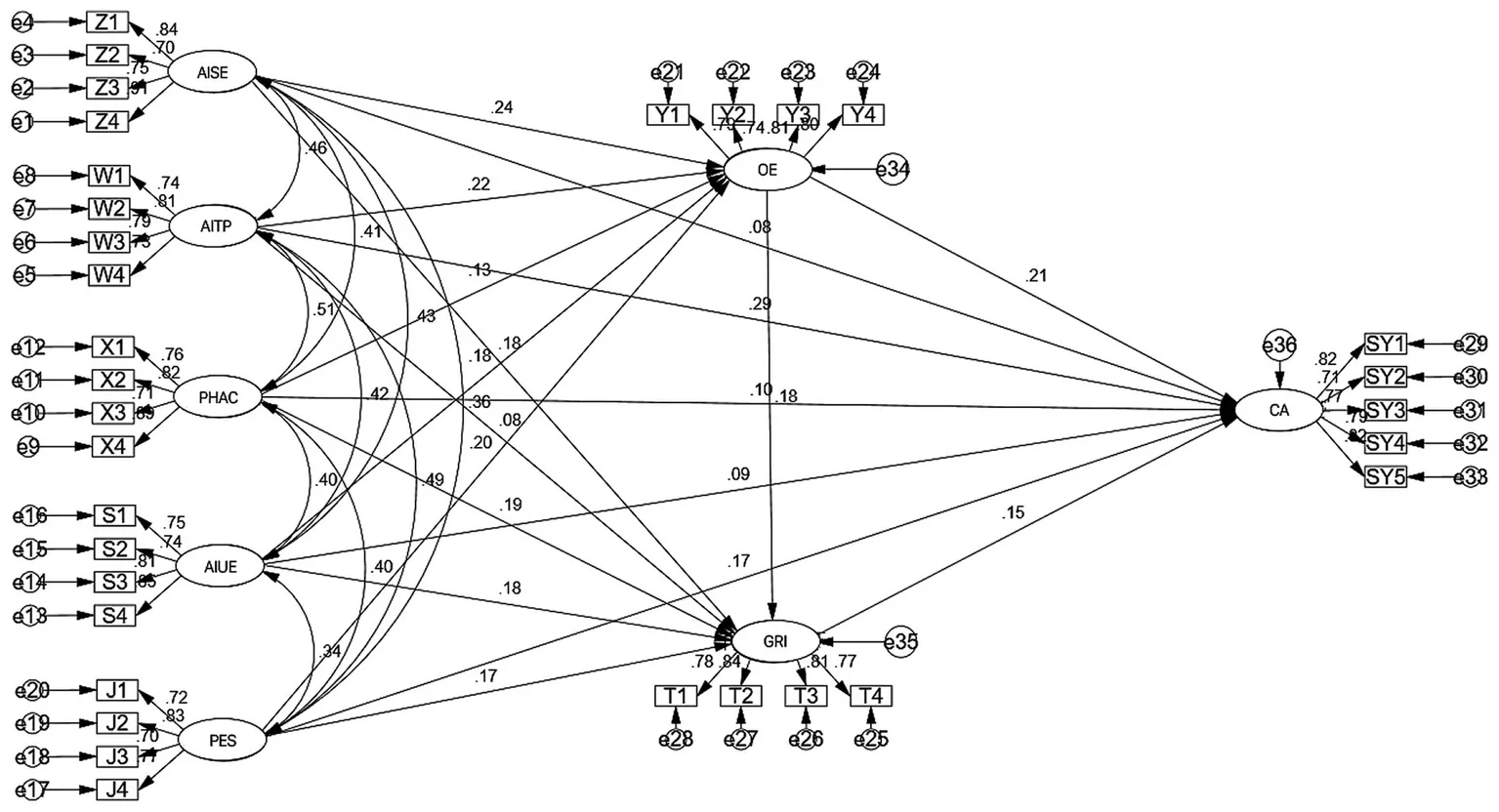
Structural model and standardised path coefficients. AISE = artificial intelligence self-efficacy; AITP = artificial intelligence threat perception; PHAC = perceived human–artificial intelligence collaboration; AIUE = artificial intelligence use engagement; PES = perceived employment support; OE = outcome expectations; GRI = goal reengagement intention; CA = career adaptability.

The SEM showed good overall fit (χ^2^/df = 1.953, RMSEA = 0.041, GFI = 0.911, CFI = 0.959, and TLI = 0.954), with all indices meeting recommended criteria. Path-test results are shown in Table 7 and Figure 1. All other 17 paths were statistically significant; the exception was the association between AI threat perception and goal reengagement intention (β = 0.080, *p* = 0.132). All five antecedents were significantly associated with outcome expectations and career adaptability. Four antecedents—all except AI threat perception—were significantly associated with goal reengagement intention. Outcome expectations were significantly associated with both goal reengagement intention (β = 0.181, *p* = 0.002) and career adaptability (β = 0.207, *p* < 0.001), while goal reengagement intention was also significantly associated with career adaptability (β = 0.149, *p* = 0.002).

**Table 7:** Structural-path analysis results.

Path	β	SE	C. R.	*p*
Artificial intelligence self-efficacy → outcome expectations	0.240	0.039	5.252	***
Artificial intelligence threat perception → outcome expectations	0.220	0.056	4.133	***
Perceived human–artificial intelligence collaboration → outcome expectations	0.133	0.041	2.824	**
Artificial intelligence use engagement → outcome expectations	0.179	0.044	3.956	***
Perceived employment support → outcome expectations	0.204	0.049	4.326	***
Artificial intelligence self-efficacy → goal reengagement intention	0.176	0.041	3.760	***
Artificial intelligence threat perception → goal reengagement intention	0.080	0.058	1.507	0.132
Perceived human–artificial intelligence collaboration → goal reengagement intention	0.185	0.043	3.940	***
Artificial intelligence use engagement → goal reengagement intention	0.182	0.046	3.957	***
Perceived employment support → goal reengagement intention	0.171	0.051	3.576	***
Outcome expectations → goal reengagement intention	0.181	0.059	3.147	**
Artificial intelligence self-efficacy → career adaptability	0.084	0.037	2.074	*
Artificial intelligence threat perception → career adaptability	0.286	0.053	6.023	***
Perceived human–artificial intelligence collaboration → career adaptability	0.104	0.038	2.539	*
Artificial intelligence use engagement → career adaptability	0.089	0.041	2.234	*
Perceived employment support → career adaptability	0.170	0.047	4.055	***
Outcome expectations → career adaptability	0.207	0.054	4.121	***
Goal reengagement intention → career adaptability	0.149	0.050	3.068	**

β denotes the standardised path coefficient. SE and C.R. are based on the corresponding unstandardised parameter estimates generated by AMOS; C.R. = critical ratio. *p < 0.05, **p < 0.01, ***p < 0.001.

### 4.4 Mediation analysis

Indirect effects were tested using 5,000 bootstrap resamples (Table 8). With the exception of the single indirect pathway linking AI threat perception to career adaptability through goal reengagement intention, the 95% confidence intervals for all indirect effects excluded zero. The indirect effects of AI self-efficacy through outcome expectations, through goal reengagement intention, and through the serial pathway involving both variables were 0.046, 0.024, and 0.006, respectively; all 95% confidence intervals excluded zero. The indirect effects of AI threat perception through outcome expectations and through the serial pathway were 0.051 and 0.007, respectively, and both 95% confidence intervals excluded zero. Its indirect effect through goal reengagement intention was 0.013 (95% CI [−0.003, 0.047]) and was not statistically significant. The indirect effects of perceived human–AI collaboration, AI use engagement, and perceived employment support through outcome expectations and goal reengagement intention were all statistically significant, but their serial indirect effects ranged only from 0.003 to 0.006 and were relatively weak. Overall, outcome expectations and goal reengagement intention exhibited statistical indirect associations of varying strength between the antecedents and career adaptability. Single indirect effects through either outcome expectations or goal reengagement intention were more pronounced, whereas serial indirect effects were generally weak.

**Table 8:** Bootstrap mediation-analysis results.

Path	Mediator	Effect	SE	95% CI	Total indirect	Total	Result
AISE → CA	OE	0.046	0.017	[0.021, 0.088]	0.075	0.152	Supported
	GRI	0.024	0.012	[0.006, 0.057]			
	Serial mediation	0.006	0.004	[0.001, 0.017]			
AITP → CA	OE	0.051	0.019	[0.022, 0.099]	0.071	0.393	Partially supported
	GRI	0.013	0.012	[−0.003, 0.047]			
	Serial mediation	0.007	0.004	[0.002, 0.020]			
PHAC → CA	OE	0.026	0.013	[0.006, 0.059]	0.055	0.153	Supported
	GRI	0.026	0.013	[0.007, 0.059]			
	Serial mediation	0.003	0.002	[0.001, 0.011]			
AIUE → CA	OE	0.038	0.018	[0.013, 0.084]	0.071	0.164	Supported
	GRI	0.028	0.014	[0.008, 0.067]			
	Serial mediation	0.005	0.003	[0.001, 0.015]			
PES → CA	OE	0.047	0.019	[0.019, 0.095]	0.082	0.271	Supported
	GRI	0.028	0.015	[0.007, 0.069]			
	Serial mediation	0.006	0.004	[0.001, 0.018]			

“Supported” indicates that all specified indirect effects were statistically significant. “Partially supported” indicates that at least one, but not all, of the specified indirect effects was statistically significant. The significance of indirect effects was determined by whether the 95% Bootstrap confidence interval excluded zero.

### 4.5 Multigroup analysis

#### 4.5.1 Measurement invariance

Before comparing the structural models for undergraduates (*n* = 429) and postgraduates (*n* = 135), configural, metric, and scalar invariance were tested sequentially. As shown in Table 9, all three models exhibited good fit. As cross-group constraints were progressively added, changes in CFI and RMSEA remained within recommended thresholds (ΔCFI < 0.01, ΔRMSEA < 0.015), and chi-square differences between adjacent models were non-significant (*p* > 0.05). Configural, metric, and scalar invariance were therefore supported across the undergraduate and postgraduate groups, indicating adequate comparability of the measurement structure, item loadings, and item intercepts and providing a measurement basis for comparing structural paths between the groups.

**Table 9:** Measurement-invariance results for undergraduates and postgraduates.

Model	χ^2^(df)	CFI	RMSEA	Δχ^2^ (Δdf)	*p*	ΔCFI	ΔRMSEA
Configural invariance	1458.497 (934)	0.952	0.032	—	—	—	—
Metric invariance	1488.663 (959)	0.952	0.031	30.166 (25)	0.218	0.000	−0.001
Scalar invariance	1519.438 (992)	0.952	0.031	30.775 (33)	0.578	0.000	0.000

#### 4.5.2 Differences in structural paths

Table 10 presents the path-difference tests. Most structural paths did not differ significantly between groups, indicating that the model relationships were generally consistent across university students at different educational stages of the school-to-work transition. Significant group differences emerged for only two paths: AI self-efficacy → outcome expectations (z = 1.996) and perceived human–AI collaboration → goal reengagement intention (z = 2.422). Both estimated coefficients were higher among postgraduates. These findings should be interpreted cautiously because the postgraduate sample was relatively small and the group sizes were unequal, which may have affected the stability of the path estimates and the statistical power of the difference tests.

**Table 10:** Differences in structural paths between undergraduate and postgraduate groups.

Path	UG β	*p*	PG β	*p*	z	Diff.
Artificial intelligence self-efficacy → outcome expectations	0.198	***	0.389	***	1.996	Yes
Artificial intelligence threat perception → outcome expectations	0.217	***	0.209	0.025	0.142	No
Perceived human–artificial intelligence collaboration → outcome expectations	0.136	0.015	0.187	0.025	0.614	No
Artificial intelligence use engagement → outcome expectations	0.196	***	0.065	0.474	−1.127	No
Perceived employment support → outcome expectations	0.197	***	0.269	0.003	0.756	No
Artificial intelligence self-efficacy → goal reengagement intention	0.196	***	0.102	0.314	−0.826	No
Artificial intelligence threat perception → goal reengagement intention	0.132	0.037	−0.091	0.353	−1.869	No
Perceived human–artificial intelligence collaboration → goal reengagement intention	0.126	0.022	0.386	***	2.422	Yes
Artificial intelligence use engagement → goal reengagement intention	0.174	***	0.183	0.053	0.141	No
Perceived employment support → goal reengagement intention	0.170	0.002	0.159	0.117	−0.099	No
Outcome expectations → goal reengagement intention	0.168	0.009	0.258	0.075	0.525	No
Artificial intelligence self-efficacy → career adaptability	0.090	0.047	0.021	0.827	−0.672	No
Artificial intelligence threat perception → career adaptability	0.273	***	0.317	0.001	0.504	No
Perceived human–artificial intelligence collaboration → career adaptability	0.142	0.002	0.009	0.924	−1.233	No
Artificial intelligence use engagement → career adaptability	0.056	0.202	0.185	0.045	1.266	No
Perceived employment support → career adaptability	0.134	0.004	0.239	0.016	0.916	No
Outcome expectations → career adaptability	0.204	***	0.268	0.059	0.318	No
Goal reengagement intention → career adaptability	0.190	***	0.003	0.981	−1.502	No

UG = undergraduate group; PG = postgraduate group; β = standardised path coefficient; *p* = within-group *p*-value; z = critical ratio for the difference between the corresponding path coefficients across groups; Diff. = between-group difference. “Yes” indicates a statistically significant difference (|z| > 1.96), whereas “No” indicates no statistically significant difference.

### 4.6 Necessary condition analysis

SEM tests average net associations between the conditions and career adaptability but cannot determine whether a condition imposes a minimum requirement that cannot be fully substituted by other conditions. NCA was therefore used to test whether each antecedent was a necessary but not sufficient condition for higher career adaptability and to identify, through bottleneck analysis, the minimum levels of conditions corresponding to different levels of career adaptability.

#### 4.6.1 Necessary-condition test results

NCA was conducted with the NCA 5.0.0 package in R 4.5.3, and results from both the ceiling envelopment–free disposal hull (CE-FDH) and ceiling regression–free disposal hull (CR-FDH) ceiling lines were reported. CE-FDH uses a non-parametric stepwise boundary without prespecifying the functional form of the ceiling relationship, whereas CR-FDH fits a linear boundary to the ceiling points and thus presents condition requirements across continuous outcome levels. To reduce dependence on a single choice of ceiling line, effect sizes and their significance were compared across both methods. Necessary-condition effects were measured using d and their statistical significance assessed through 10,000 permutation tests. These tests randomly permuted the case-level correspondence between a condition and the outcome, repeatedly estimated the necessary-condition effect, and compared the observed effect with the permutation distribution to obtain a *p* value. Following Dul (2016), effect sizes were classified as small when d < 0.10, medium when 0.10 ≤ d < 0.30, large when 0.30 ≤ d < 0.50, and very large when d ≥ 0.50.

As Table 11 shows, the necessity effect sizes of the antecedent conditions ranged from 0.011 to 0.063 and were all small. Perceived employment support, outcome expectations, and goal reengagement intention were statistically significant under both ceiling-line methods. AI threat perception was marginally significant under CE-FDH and significant under CR-FDH. The significance of perceived human–AI collaboration and AI use engagement varied by ceiling-line method, while AI self-efficacy was non-significant under both methods. Although goal reengagement intention had the largest relative effect size, it remained below 0.10. The results therefore identified no single necessary condition that had a substantial practical effect and remained stable across both ceiling-line methods.

**Table 11:** Necessary condition analysis results.

Condition	Method	Accuracy	Ceiling zone	Effect size	*p*
Artificial intelligence self-efficacy	CE-FDH	100.0%	0.250	0.016	0.271
	CR-FDH	99.8%	0.168	0.011	0.219
Artificial intelligence threat perception	CE-FDH	100.0%	0.350	0.022	0.050
	CR-FDH	99.5%	0.231	0.014	0.028
Perceived human–artificial intelligence collaboration	CE-FDH	100.0%	0.400	0.025	0.052
	CR-FDH	99.7%	0.323	0.020	0.023
Artificial intelligence use engagement	CE-FDH	100.0%	0.950	0.059	0.017
	CR-FDH	100.0%	0.475	0.030	0.055
Perceived employment support	CE-FDH	100.0%	0.550	0.034	0.028
	CR-FDH	99.7%	0.424	0.026	0.021
Outcome expectations	CE-FDH	100.0%	0.500	0.031	0.020
	CR-FDH	99.8%	0.339	0.021	0.021
Goal reengagement intention	CE-FDH	100.0%	1.000	0.063	***
	CR-FDH	99.4%	0.773	0.048	***

*p < 0.05, **p < 0.01, ***p < 0.001.

#### 4.6.2 Bottleneck analysis

A bottleneck analysis based on the CR-FDH ceiling line was conducted to show the minimum condition levels corresponding to different levels of career adaptability (Table 12). At career-adaptability levels of 80% or below, most conditions exhibited no clear minimum requirement, and only AI use engagement showed a low bottleneck level. At a career-adaptability level of 90%, all seven conditions began to show minimum requirements, with goal reengagement intention having the highest requirement (19.6%). At 100% career adaptability, the bottleneck level for goal reengagement intention increased to 69.2%, markedly higher than those of the other conditions. In conjunction with the preceding finding that all effect sizes were below 0.10, these bottleneck values primarily reflect relative lower bounds within the sample and should not be interpreted as strict necessary conditions with substantial effects.

**Table 12:** Necessary condition analysis bottleneck levels (%).

Career-adaptability level	AISE	AITP	PHAC	AIUE	PES	OE	GRI
0	NN	NN	NN	NN	NN	NN	NN
10%	NN	NN	NN	0.3	NN	NN	NN
20%	NN	NN	NN	1.0	NN	NN	NN
30%	NN	NN	NN	1.6	NN	NN	NN
40%	NN	NN	NN	2.3	NN	NN	NN
50%	NN	NN	NN	3.0	NN	NN	NN
60%	NN	NN	NN	3.6	NN	NN	NN
70%	NN	NN	NN	4.3	NN	NN	NN
80%	NN	NN	NN	4.9	NN	NN	NN
90%	5.2	0.5	10.0	5.6	13.2	10.4	19.6
100%	11.5	28.3	22.3	6.2	28.0	24.0	69.2

NN indicates that no identifiable necessary bottleneck level exists at the corresponding level of career adaptability.

### 4.7 Fuzzy-set qualitative comparative analysis

SEM can estimate average net associations and NCA can test necessity constraints for individual conditions, but neither readily reveals the conjunctural, substitutive, and compensatory relationships among multiple conditions. This study therefore used fsQCA as an exploratory set-relational approach to identify multiple configurations of conditions associated with higher career adaptability. In the present cross-sectional study, fsQCA was used to examine conjunctural relationships, equifinality, and configurational asymmetry, thereby complementing linear net-effect analysis.

#### 4.7.1 Variable calibration and truth-table settings

AI self-efficacy, AI threat perception, perceived human–AI collaboration, AI use engagement, perceived employment support, outcome expectations, and goal reengagement intention were specified as conditions, with career adaptability as the outcome set. Because the scales had no external theoretical cut-off values that could be applied directly, distribution-based direct calibration was used. The 90th, 50th, and 10th percentiles of each variable were set as the thresholds for full membership, the crossover point, and full non-membership, respectively. Cases with a calibrated membership score of exactly 0.500 were adjusted to 0.501 to avoid ambiguity at the crossover point (Gligor and Bozkurt, 2020). Before the sufficiency analysis, a consistency threshold of 0.90 was used to assess the necessity of individual conditions. The consistency of every antecedent condition and its negation was below 0.90, indicating that no single condition was necessary for higher career adaptability, consistent with the NCA results.

The seven conditions produced 128 potential truth-table configurations. To exclude incidental configurations lacking an empirical basis while preserving configurational diversity, the case-frequency threshold was set at 3, the minimum sufficiency-consistency threshold at 0.80, and the proportional reduction in inconsistency (PRI) threshold at 0.75. The latter excluded ambiguous configurations exhibiting high consistency with both the outcome set and its negation (Greckhamer et al., 2013). Among truth-table rows meeting the frequency threshold, the lowest consistency entering the minimisation analysis for the baseline model was 0.893256. Rows meeting the frequency threshold but failing either the consistency cut-off or the PRI threshold were coded 0 and excluded from sufficiency minimisation, but their cases were not deleted. Core and peripheral conditions were identified by comparing the parsimonious and intermediate solutions: conditions appearing in both were classified as core, and those appearing only in the intermediate solution as peripheral.

#### 4.7.2 Configurational results

The intermediate solution for the baseline model identified 23 configurations. Six representative configurations were selected for presentation in the main text on the basis of configuration consistency, raw coverage, and differences in condition structure (Table 13). P2, with AI self-efficacy and outcome expectations as core conditions, represents a cognition-dominant configuration. P4, with AI threat perception and goal reengagement intention as core conditions, represents a risk-related configuration. P1 and P3 both feature AI threat perception and AI use engagement as principal core conditions and represent engagement-related configurations. P5 and P6 demonstrate potentially compensatory combinations under the absence of some conditions.

**Table 13:** Fuzzy-set qualitative comparative analysis of configurational associations with career adaptability.

Condition	P1	P2	P3	P4	P5	P6
Artificial intelligence self-efficacy	○	●			○	⊙
Artificial intelligence threat perception	●		●	●	●	
Perceived human–artificial intelligence collaboration	●	○			⊙	○
Artificial intelligence use engagement	●		●			⊙
Perceived employment support		○	●	○	○	⊙
Outcome expectations		●	○	○	⊙	⊙
Goal reengagement intention				●		●
Raw coverage	0.420	0.443	0.426	0.447	0.239	0.163
Unique coverage	0.004	0.012	0.011	0.009	0.008	0.003
Consistency	0.871	0.886	0.883	0.875	0.916	0.915
Solution coverage	0.799					
Solution consistency	0.761					

● indicates the presence of a core condition; ○ indicates the presence of a peripheral condition; and ⊙ indicates the absence of a peripheral condition.

The consistency of the six representative configurations ranged from 0.871 to 0.916, indicating relatively consistent set relationships between each individual configuration and higher career adaptability. Overall solution coverage was 0.799 and overall solution consistency was 0.761. The truth-table row consistency threshold and overall solution consistency represent different aspects of the analysis: the former determines whether an individual truth-table row enters logical minimisation, whereas the latter reflects the consistency between the union of all configurations and the outcome set. Although the overall solution consistency was below 0.80, the representative configurations individually showed comparatively high consistency and captured recurring combinations of cognitive, behavioural, and contextual conditions within the current sample. Their interpretive value therefore lies in revealing configurational diversity, including possible substitution and compensation among conditions. Nevertheless, these findings are interpreted as exploratory configurational associations rather than as confirmed sufficient pathways.

#### 4.7.3 Robustness test

To test the robustness of the fsQCA findings, the calibration anchors, frequency threshold, and PRI threshold were held constant while the minimum sufficiency-consistency criterion was increased from 0.80 to 0.90. The actual consistency cut-off consequently increased from 0.893 to 0.922, the number of intermediate-solution configurations decreased from 23 to 16, overall coverage changed from 0.799 to 0.759, and overall consistency increased from 0.761 to 0.788. Under the stricter consistency criterion, the principal configurations involving AI threat perception, AI use engagement, and goal reengagement intention were retained, while only some peripheral configurations were excluded. Although overall solution consistency remained below 0.80, the principal combinations of conditions did not change substantively. The findings therefore demonstrated a degree of configurational stability, although some peripheral configurations remained sensitive to the consistency threshold. Accordingly, the robustness results support the exploratory value of the principal configurational associations rather than establishing the sufficiency of the overall solution.

## 5 Discussion, implications, and future directions

### 5.1 Discussion of the findings

#### 5.1.1 Relational structure of cognitive appraisal and action regulation

Drawing on the SEM and mediation-analysis results, this study examined, from a variable-relational perspective, the relational structure of career adaptability among university students in the school-to-work transition in the AI employment context. Overall, the findings show that multidimensional factors are not associated with career adaptability solely through direct relationships. Rather, they form a relational structure that also involves variables representing cognitive appraisal and action regulation.

At the cognitive level, AI self-efficacy, AI threat perception, perceived human–AI collaboration, AI use engagement, and perceived employment support were all significantly associated with outcome expectations. This finding accords with the SCCT proposition that cognitive appraisal is a precursor to behaviour (Lent and Brown, 2013) and with the expectancy–value proposition that individuals develop behavioural propensities on the basis of anticipated benefits (Cheng, 2025). Beyond studies that explain career behaviour using a single variable, the present findings further indicate that individuals in the AI context are likely to form an overall judgement of their future career development from multidimensional information concerning capabilities, the environment, and resources.

At the action level, both outcome expectations and goal reengagement intention were significantly associated with career adaptability, demonstrating the importance of cognitive appraisal and action regulation in explaining career adaptability. This finding is consistent with previous evidence that positive expectations help sustain behavioural engagement. However, the present study also found that the strength of the indirect effects varied across pathways and that the effects of the serial pathways were particularly small. Although cognitive and behavioural variables are associated, the strength of their relationships may therefore depend on multiple factors rather than following a single stable pathway.

Notably, AI threat perception played differentiated roles in the model. It was significantly associated with outcome expectations and career adaptability but not with goal reengagement intention. This finding is not entirely consistent with studies arguing that threat directly inhibits behavioural engagement, and it did not support the hypothesised positive association between AI threat perception and goal reengagement intention. The present data provide no evidence of a significant association between AI threat perception and goal reengagement intention, indicating that risk perception does not necessarily correspond to a willingness to adjust goals. The finding may also reflect the study context. In China’s competitive employment environment, university students in the school-to-work transition may experience a degree of motivation to act or prepare adaptively—through advance planning or capability development, for example—alongside their perception of employment risks. This may account for the overall positive association between AI threat perception and career adaptability.

This interpretation must nevertheless remain within the limits of the cross-sectional design. The positive association may also reflect a reverse relationship: individuals with greater career adaptability may be more sensitive to labour-market change and report higher AI threat perception because they monitor industry and job information more proactively. Variables omitted from the model, such as willingness to explore careers, capacity to search for career information, or future time perspective, may also simultaneously increase risk perception and career preparedness, producing a positive association between AI threat perception and career adaptability. Although the causal direction remains unclear, the results indicate at minimum that AI threat perception in the school-to-work transition is not associated exclusively with poorer adaptation; it may coexist with career vigilance and adaptive preparation. This provides new empirical insight into the complex relationship between risk perception and career adaptation under technological pressure.

#### 5.1.2 Group heterogeneity and differences in structural paths

The multigroup analysis showed that, once measurement invariance had been substantially established, the overall structural model did not differ significantly between undergraduates and postgraduates, and most paths were consistent across the two groups. Within this sample, the pattern of relationships underlying career adaptability in the AI context thus displayed a degree of consistency across educational stages. This broadly accords with previous findings that career-related cognitive and behavioural mechanisms are relatively stable across student groups. Only a few relationships exhibited statistically significant differences. The coefficient for the path from AI self-efficacy to outcome expectations was larger among postgraduates, as was that from perceived human–AI collaboration to goal reengagement intention. These differences concerned pathway strength, however, their statistical significance was close to the threshold, and their stability should be viewed cautiously given the unequal group sizes. Previous research suggests that educational stages may differ in career cognition and information use: postgraduates may have relative advantages in professional experience and information integration, whereas undergraduates may rely more heavily on external information when making career judgements. The present evidence is insufficient to establish these mechanisms as stable explanations within this model; the differences should therefore be treated as descriptive findings rather than definitive conclusions. The remaining paths did not differ significantly between groups, indicating relatively consistent relationships involving AI threat perception, AI use engagement, perceived employment support, and the mediating variables. This finding lends some support to the generality of career-adaptation mechanisms across student populations, while also suggesting that educational stage may not be the only or principal source of variation in these relationships.

#### 5.1.3 Multiple pathways and constraint mechanisms

Combining the NCA and fsQCA findings with the structural analysis provides a further account of career adaptability in terms of condition constraints and configurational relationships. The NCA effect sizes were generally small, and most conditions did not meet the criterion for a strong necessary condition. No single factor therefore imposed a stable necessity constraint on career adaptability within this sample. This is consistent with the joint, multivariable associations observed in the SEM.

The exploratory fsQCA identified multiple configurations associated with higher career adaptability.

First, the risk-related configuration combined AI threat perception and goal reengagement intention as core conditions with peripheral conditions including perceived employment support and outcome expectations. It thus reflected the conjunction of risk perception, goal adjustment, and behavioural engagement. AI-related uncertainty was not associated only with poorer adaptation; under particular configurations, it coexisted with higher career adaptability. Second, the cognition-dominant configuration combined AI self-efficacy and outcome expectations as core conditions, reflecting a configuration of capability cognition and positive expectations. When individuals can form clear judgements based on their capabilities and maintain positive expectations, they may exhibit higher career adaptability even when external support is relatively limited. Finally, the engagement-related compensatory configuration combined AI threat perception and AI use engagement as core conditions. When some cognitive or resource conditions are relatively weak, greater actual engagement may combine with other conditions to compensate for those limitations. Behavioural engagement thus represents not only a state of action in career adaptation but, under certain conditions, an important component of configurations associated with higher career adaptability.

### 5.2 Research implications

#### 5.2.1 Theoretical implications

First, this study situates SCCT within an employment environment in which AI has both substitutive and collaborative attributes, extending the theory’s explanatory reach to a complex technological context. International research has often separately emphasised the anxiety, avoidance, and use hesitation induced by AI-related threats (Kitchens and Meier, 2026; Yin, 2025), or the capability enhancement and adaptive opportunities produced by human–AI collaboration and technology-related engagement (Lu and Kim, 2025; Li and Li, 2026). The present findings show that career cognition in the AI employment context cannot be reduced to a threat–opportunity dichotomy; it represents an integrated appraisal of technological risks, capability resources, and development possibilities. In particular, the positive association between AI threat perception and career adaptability suggests that technological threat is not necessarily an exclusively inhibitory factor and may coexist with career vigilance and adaptive preparation.

Second, the study distinguishes cognitive appraisal from action regulation in career adaptation. Outcome expectations capture individuals’ judgements of future career outcomes, whereas goal reengagement intention captures redirection after an existing goal is obstructed. The two do not form a fixed linear sequence but participate in career adaptation together with capability beliefs, technology-related engagement, and contextual support and may exhibit substitution and compensation. The study therefore both refines SCCT’s account of the relationship between career cognition and action and extends CCT’s view that career adaptation depends on configurations of multiple psychosocial resources. The exploratory findings suggest that career adaptability may be associated with different resource configurations rather than with a single dominant pattern.

#### 5.2.2 Practical implications

First, university career guidance should provide coordinated support for cognitive appraisal and action regulation. Both outcome expectations and goal reengagement intention were significantly associated with career adaptability, suggesting that employment information or AI-skills training alone may not address the different needs involved in students’ career adaptation. Universities could use courses on AI-related employment trends, career-scenario simulations, and data-informed employment-analysis platforms to help students form reasonable outcome expectations in light of their capabilities and the employment environment (Rathee and Mittal, 2024). They could also introduce stage-based goal management and process-feedback mechanisms, such as phased assessments of employment plans and tracking of practical tasks, to help students translate career judgements into sustained action. Policymakers could promote information linkages among demand for AI-related jobs, occupational capability standards, university curricula, and internship programmes. For example, regional or industry-specific mechanisms could regularly publish and update information about demand for AI-related positions and the associated capability standards, providing more timely labour-market evidence for university career guidance, career counselling, and AI-literacy programmes.

Second, although most relational structures were consistent across undergraduates and postgraduates, a few pathways differed in strength, suggesting that universities may adjust the emphasis of support while maintaining common educational content. AI-literacy programmes for undergraduates could strengthen career cognition, foundational AI literacy, and technology-application training to help students assess the career environment and their own capabilities. For postgraduates, universities could further integrate professional competence with human–AI collaborative practice—for example, through participation in research projects, university–industry collaborations, and specialised AI-application activities—so that students can apply their professional experience to career judgement and action adjustment (Segbenya et al., 2023). Employers could likewise design progressively demanding tasks for internship and recruitment activities according to students’ stage of education. Because most paths did not differ significantly between groups, however, this differentiation should adjust the emphasis of support rather than create entirely separate training programmes for undergraduates and postgraduates.

Third, the findings suggested multiple possible resource configurations associated with career adaptability, while no single necessary condition had a substantial practical effect. Although goal reengagement intention was relatively prominent in the bottleneck analysis for high career adaptability, its overall necessity effect remained small. University and employer interventions should therefore extend beyond the provision of a single resource to encompass practical opportunities, process feedback, and support for goal adjustment. Universities could provide more practice in authentic contexts, including AI-project training and joint university–industry programmes, while creating a learning environment that permits experimentation and adjustment (Sozon et al., 2026). Employers could use progressively demanding tasks and continuous feedback to help interns or graduates accumulate experience of human–AI collaboration. Universities and employers could also provide multiple career-preparation pathways for students with different resource profiles through practice certification, capability assessment, and the sharing of employment information (Liu and Mei, 2026).

### 5.3 Limitations and future directions

Although this study systematically analysed the relational structure and configurational associations of career adaptability among university students in the school-to-work transition through an integrated multi-method framework, it has several limitations. First, the study used cross-sectional questionnaire data. The results therefore primarily represent statistical associations and cannot establish the temporal ordering of outcome expectations, goal reengagement intention, and career adaptability or capture dynamic changes in career adaptability. The SEM also did not include demographic variables such as gender, discipline, and type of institution as controls, and potential confounding cannot be completely excluded. Future research could use longitudinal or multiwave designs and a more comprehensive set of controls to examine the direction of the relationships. Second, convenience sampling limits the generalisability of the findings. The postgraduate sample was relatively small and group sizes across educational stages were unequal, which may have affected the stability of path estimates and the statistical power of group-difference tests. Future studies could replicate the analyses with larger and more balanced samples. Third, the study examined career adaptability primarily from the perspective of individual university students and did not incorporate assessments by employers or other hiring organisations. Future research could introduce data on job requirements and organisational contexts and extend the analytical framework from the perspectives of supply–demand fit or cross-level interactions. Fourth, although the representative configurations exhibited relatively high individual consistency and the principal patterns were retained in the robustness analysis, the overall solution consistency remained comparatively limited. The configurational findings should therefore be interpreted as exploratory associations observed within the current sample rather than as definitive sufficient pathways. Future research could examine the stability of these patterns using independent samples, alternative calibration and threshold settings, and longitudinal or multiwave designs.

## 6 Conclusion

The central contribution of this study is not the confirmation of the importance of any single factor for career adaptability, but the revelation of the complex structure of career adaptation in the AI employment context. For individuals in the school-to-work transition, AI is neither solely an employment threat nor solely a development opportunity. Its career-related meaning is closely associated with individuals’ integrated appraisals of technological risks, their own capabilities, possibilities for human–AI collaboration, actual use experience, and external support. The positive association between AI threat perception and career adaptability further indicates that risk perception does not necessarily correspond to poorer adaptation and may coexist with career vigilance and preparedness. Understanding the implications of AI for career development thus requires moving beyond a binary judgement of facilitation versus inhibition to examine how individuals form career judgements and adaptive preparation in an environment where pressure and opportunity coexist. The overall relational structure was relatively stable across educational stages, with only a few relationships differing in strength. Undergraduates and postgraduates therefore share a similar structure of career adaptation, although some relationships may vary in emphasis according to professional experience and the context of human–AI collaboration. At the same time, the necessity and configurational analyses indicated that higher career adaptability does not depend on a single necessary condition with a substantial practical effect. The exploratory configurational findings suggest that higher career adaptability may be associated with cognition-oriented, risk-related, and engagement-related configurations, reflecting possible substitution and compensation among different resources.

Overall, this study extends research on career adaptation in the AI employment context from a singular focus on coping with technological threat to an integrated explanation of how individuals combine risk perceptions, capability resources, and action regulation. It further suggests that similar technological changes and employment pressures may be associated with different patterns of career adaptation. This conclusion not only refines the application of SCCT and CCT in complex technological environments but also shows that, as AI continues to alter job structures, skill requirements, and occupational boundaries, the key to career adaptation is not to eliminate uncertainty but to understand how individuals respond to it through different resource structures. The study thus provides a more contextualised and multi-method explanatory framework for understanding the career development of university students in the school-to-work transition as AI reshapes the labour market.

## Data Availability

The raw data supporting the conclusions of this article will be made available by the authors, without undue reservation.
